# Novel Phenolic Inhibitors of Small/Intermediate-Conductance Ca^2+^-Activated K^+^ Channels, KCa3.1 and KCa2.3

**DOI:** 10.1371/journal.pone.0058614

**Published:** 2013-03-14

**Authors:** Aida Oliván-Viguera, Marta Sofía Valero, María Divina Murillo, Heike Wulff, Ángel-Luis García-Otín, José-Miguel Arbonés-Mainar, Ralf Köhler

**Affiliations:** 1 Aragon Institute of Health Sciences I+CS, Zaragoza, Spain; 2 Department of Cardiovascular and Renal Research, Institute of Molecular Medicine, University of Southern Denmark, Odense, Denmark; 3 GIMACES, Faculty of Health Sciences, Universidad San Jorge, Villanueva de Gállego, Spain; 4 Department of Pharmacology and Physiology, Facultad de Veterinaria, Universidad de Zaragoza, Zaragoza, Spain; 5 Department of Pharmacology, University of California Davis, Davis, California, United States of America; 6 GIPASC, Laboratorio de Investigación Molecular, Hospital Universitario Miguel Servet, Zaragoza, Spain; 7 Adipocyte and Fat Biology Laboratory, Hospital Universitario Miguel Servet, Zaragoza, Spain; 8 Fundación Agencia Aragonesa para la Investigación y Desarrollo (ARAID), Zaragoza, Spain; University of Houston, United States of America

## Abstract

**Background:**

KCa3.1 channels are calcium/calmodulin-regulated voltage-independent K^+^ channels that produce membrane hyperpolarization and shape Ca^2+^-signaling and thereby physiological functions in epithelia, blood vessels, and white and red blood cells. Up-regulation of KCa3.1 is evident in fibrotic and inflamed tissues and some tumors rendering the channel a potential drug target. In the present study, we searched for novel potent small molecule inhibitors of KCa3.1 by testing a series of 20 selected natural and synthetic (poly)phenols, synthetic benzoic acids, and non-steroidal anti-inflammatory drugs (NSAIDs), with known cytoprotective, anti-inflammatory, and/or cytostatic activities.

**Methodology/Principal Findings:**

In electrophysiological experiments, we identified the natural phenols, caffeic acid (EC50 1.3 µM) and resveratrol (EC50 10 µM) as KCa3.1 inhibitors with moderate potency. The phenols, vanillic acid, gallic acid, and hydroxytyrosol had weak or no blocking effects. Out of the NSAIDs, flufenamic acid was moderately potent (EC50 1.6 µM), followed by mesalamine (EC50≥10 µM). The synthetic fluoro-trivanillic ester, 13b ([3,5-bis[(3-fluoro-4-hydroxy-benzoyl)oxymethyl]phenyl]methyl 3-fluoro-4-hydroxy-benzoate), was identified as a potent mixed KCa2/3 channel inhibitor with an EC50 of 19 nM for KCa3.1 and 360 pM for KCa2.3, which affected KCa1.1 and Kv channels only at micromolar concentrations. The KCa3.1/KCa2-activator SKA-31 antagonized the 13b-blockade. In proliferation assays, 13b was not cytotoxic and reduced proliferation of 3T3 fibroblasts as well as caffeic acid. In isometric vessel myography, 13b increased contractions of porcine coronary arteries to serotonin and antagonized endothelium-derived hyperpolarization-mediated vasorelaxation to pharmacological KCa3.1/KCa2.3 activation.

**Conclusions/Significance:**

We identified the natural phenols, caffeic acid and resveratrol, the NSAID, flufenamic acid, and the polyphenol 13b as novel KCa3.1 inhibitors. The high potency of 13b with pan-activity on KCa3.1/KCa2 channels makes 13b a new pharmacological tool to manipulate inflammation and cancer growth through KCa3.1/KCa2 blockade and a promising template for new drug design.

## Introduction

The intermediate-conductance Ca^2+^-activated K^+^ channel, KCa3.1, belongs to the gene family of calcium/calmodulin-regulated and voltage-independent K^+^ channels (KCa2.1/2.2/2.3 and KCa3.1) [1], [2] and contributes to cellular functions by producing membrane hyperpolarization and thus regulating intracellular Ca^2+^ signaling. KCa3.1 channels are expressed in red and white blood cell lineages [3], [4], [5], epithelia [6], [7] and endothelia [8], [9] where KCa3.1 contributes to volume regulation, clonal expansion, fluid secretion, and vasodilatation. From the pathophysiological perspective, up-regulation of KCa3.1 expression is a common feature of activated and proliferating cells like T-cells [5], endothelial cells [10], neointimal smooth muscle cells [11], [12], fibroblasts [13], [14], and some cancer types such as glioblastomas [15], [16], [17]. In these tissues, KCa3.1 channels have been suggested to promote immune responses [5], [18], angiogenesis [10], atherosclerosis [19], arterial restenosis [11], [20], fibrosis [14], and cancer growth [15], thus rendering the channel a promising drug target in these disease states. Accordingly, a number of studies by several groups showed that small molecule inhibitors of KCa3.1 such as TRAM-34 and ICA-17043 (Senicapoc) were to some degree efficient in halting such disease processes in animal models (for review see [18], [21]).

Here, we screened for negative gating modulators (i.e. non-pore inhibitors) as alternatives to the existing pore blockers [18] and started by testing “privileged” drug-like structures such as simple natural phenolic and benzoic molecules, synthetic non-steroidal anti-inflammatory drugs (NSAIDs) and more complex synthetic polyphenols, with reported cytoprotective, anti-inflammatory, analgesic, and/or cytostatic activities (for structures see Figure S1). We next tested whether the most potent novel KCa3.1-blocking compound identified in the present study would affect two different KCa3.1-mediated cellular functions: 1) in vitro proliferation of fibroblasts and 2) ex-vivo endothelial vasodilator function.

The electrophysiological screening of natural and synthetic compounds revealed that the natural phenols, caffeic acid and resveratrol, as well as the NSAID, flufenamic acid, are moderately potent KCa3.1 inhibitors. The synthetic tri-fluoro trivanillic ester ([3,5-bis[(3-fluoro-4-hydroxy-benzoyl)oxymethyl]phenyl]methyl 3-fluoro-4-hydroxy-benzoate, 13b) with a previously reported pan-anti-kinase activity at low micromolar concentrations [22], [23] was found to be a potent KCa3.1 and KCa2.3 inhibitor with EC50s in the lower nanomolar (KCa3.1) or picomolar range (KCa2.3) that inhibited fibroblast proliferation and reduced endothelium-derived hyperpolarization-mediated relaxations of porcine coronary arteries.

## Materials and Methods

### Cell Lines

3T3 fibroblasts (3T3-L1, mouse embryonic fibroblast, ref# CL-173, American Type Culture Collection, Rockville, MD, USA), U251 glioblastoma cells, porcine coronary artery endothelial cells (PCAEC), hKCa3.1-HEK293 cells [24], hKv1.2-B82 cells (murine fibroblast cell line) [25], hKv1.3-L929 cells (fibroblast cell line from murine lung, [26], hERG-HEK293 and hKCa2.3-COS7 cells [27] were grown in culture dishes containing Dulbecco’s Modified Eagle Medium (DMEM) supplemented with 10% fetal calf serum (FCS) and penicillin/streptomycin (all from Biochrom KG, Berlin, Germany). With the exception of hKCa2.3-COS7 cells, the above mentioned cell lines and cell lines stably expressing cloned human channels were generous gifts from several sources: The 3T3 fibroblasts were obtained from MJ Moreno-Aliaga, Department of Physiology and Nutrition, School of Pharmacy, University of Navarra, Pamplona, Spain. The hKCa3.1 were obtained from Khaled Houamed, University of Chicago (Chicago, IL). HERG expressed in HEK-293 cells were obtained from Craig January, University of Wisconsin, Madison, WI). U251 cells were obtained from Pilar Martín Duque (Aragon Institute of Health Sciences I+CS and ARAID, Zaragoza, Spain). Isolation of PCAEC: Hearts were kindly provided by the local abattoir (Matadero Mercazaragoza). Left anterior descending and posterior descending coronary arteries (CA) were carefully dissected and cleaned of connective tissue and fat. CA were cut open longitudinally and incubated in trypsin/EDTA (0.25%/0,02%) in PBS without Ca^2+^/Mg^2+^ (Biochrom KG) for 30 min. Thereafter the luminal surface was carefully scrapped with a pipette tip and cells were aspirated and transferred into culture dishes containing cover slips. Cells were used for patch-clamp experiments within 24 hrs.

Prior to electrophysiological studies, cells were trypsinized and seeded on cover slips in a NaCl solution composed of (mM): 140 NaCl, 5 KCl, 1 MgSO_4_, 1 CaCl_2_, 10 glucose and 10 HEPES (adjusted to pH 7.4 with NaOH). Cells were allowed to settle down for 2–4 hrs and used for electrophysiological measurements within 6 hrs.

### Patch-clamp Electrophysiology

Whole-cell membrane currents were recorded using an EPC10-USB patch-clamp amplifier (HEKA Electronics, Germany) using voltage-ramps (−100 to 100 mV, 1 sec) followed by a single 0 mV pulse for 1 sec (for quantifying the amplitude of K^+^-outward currents) and analyzed with the Patchmaster™ software. Human ERG currents were recorded with a pre-pulse to −80 mV (for 1 sec), followed by a depolarizing pulse to +30 mV (1 sec duration) and a repolarizing pulse to −40 mV (500 msec duration) to measure amplitudes of tail currents. Leak subtraction was not performed during data acquisition, but “ohmic” leak of up to 6 nS was subtracted at the time of data analysis if appropriate. For activation of KCa currents, cell were dialyzed with a KCl-pipette solution containing 1 µM [Ca^2+^]_free_ (in mM): 140 KCl, 1 MgCl_2_, 2 EGTA, 1.71 CaCl_2_, and 5 HEPES (adjusted to pH 7.2 with KOH). The pipette solution used for measuring Kv channels contained 100 nM [Ca^2+^]_free_ (2 mM EGTA, 0.7 mM CaCl_2_). The composition of the NaCl bath solution was as stated above. For single-channel recordings in inside-out patches from hKCa3.1-overexpressing HEK293 cells, we used an Axopatch amplifier (Axon Instruments) and post-filtered the data at 100 Hz. The bath solution contained 0.5 µM [Ca^2+^]_free_ (in mM): 140 KCl, 1 MgCl_2_, 2 EGTA, 1.48 CaCl_2_, and 5 HEPES (adjusted to pH 7.2 with KOH). The bath solution stated above was used as pipette solution. For blocking experiments, we used the selective KCa3.1 blocker TRAM-34 [5] (1 µM) and tested phenolic and poly-phenolic compounds as stated in table 1 to evaluate potential blocking efficiency. For calculation of EC50 values, data points were fitted using the equation: y = A2+ (A1–A2)/(1+ (x/x0)∧p) or the Boltzmann equation: y = A2+ (A1–A2)/(1+ exp((x-x0)/dx)), depending on which equation gave the better fit. For further activation or recovery of currents, the KCa3.1/KCa2.X activator naphtho[1,2-*d*]thiazol-2-ylamine (SKA-31, 1 µM) was added to the bath solution.

**Table 1:** Inhibitory effects of natural and synthetic (poly)phenols, synthetic benzoic acids, and non-steroidal anti-inflammatory drugs on KCa3.1 channels.

Compound	% of control at					
	1 µM	10 µM		50 µM		
**Hydroxytyrosol** ((2-hydroxyethyl)benzene-1,2-diol)	117±9	108±15		NT		
**Gallic acid** (3,4,5-trihydroxybenzoic acid)	NT	101±6		NT		
**Vanillic Acid** (4-hydroxy-3-methoxybenzoic acid)	NT	78±11		46±12		
**Caffeic Acid** ((E)-3-(3,4-dihydroxyphenyl)propenoic acid)	59±6	22±11		0.8±0.3		
**Ferulic Acid** ((E)-3-(4-hydroxy-3-methoxyphenyl)propenoic acid)	NT	87±8		32±4		
**Resveratrol** (5-[(E)-2-(4-Hydroxyphenyl)vinyl]benzene-1,3-diol)	NT	44±1		12±3		
**Caffeic acid phenethyl ester** (2-phenylethyl (E)-3-(3,4-dihydroxyphenyl)propenoate)	NT	94±6		NT		
**1,2,3,4,6-pentagalloyl glucose** ([(2*S*,3*R*,4*S*,5*R*,6*R*)-2,3,5-tris[(3,4,5-trihydroxybenzoyl)oxy]-6-[(3,4,5-trihydroxybenzoyl)oxymethyl]oxan-4-yl] 3,4,5-trihydroxybenzoate)	NT	102±6		NT		
**2-acetyloxybenzoic acid** (Aspirin™)	NT	108±5		104±7		
**Mesalamine** (5-amino-2-hydroxybenzoic acid)	72±6	53±7		46±3		
**Mefenamic Acid** (2-((2,3-dimethylphenyl)amino)benzoic acid)	117±4	136±28		NT		
**Niflumic Acid** (2-((3-(trifluoromethyl)phenylamino) nicotinic acid)	NT	106±7		81±4		
**Flufenamic Acid** (2-(3-(trifluoromethyl)phenylamino) benzoic acid)	65±4	13±5		2.5±0.3		
**Modafinil** (2-benzhydrylsulfinylacetamide)	NT	33±6		NT		
**Pirfenidone** (methyl-1-phenylpyridin-2(1H)-one)	NT	98±5		99±14		
**13a** ([3,5-bis[(4-hydroxy-3-methoxy-benzoyl)oxymethyl]phenyl]methyl4-hydroxy-3-methoxy-benzoate)	78±6	NT		NT		
**13b** ([3,5-bis[(3-fluoro-4-hydroxy-benzoyl)oxymethyl]phenyl]methyl3-fluoro-4-hydroxy-benzoate)	4±1	NT		NT		
**13c** ([3,5-bis[(3-chloro-4-hydroxy-benzoyl)oxymethyl]phenyl]methyl3-chloro-4-hydroxy-benzoate)	61±17	NT		NT		
**TRAM-34** (1-[(2-chlorophenyl) (diphenyl)methyl]-1*H*-pyrazole)	1±1	NT		NT		
**SKA-31** (naphtho[1,2-*d*] [1], [3]thiazol-2-amine)	329±76 (activation)		NT		NT	

NT, not tested; data are given as mean ± SEM, n ≥3.

### Cell Proliferation Assays

Cell proliferation was spectrophotometrically assessed as described previously [28] with some modifications. Briefly, 3T3 fibroblasts (1500 cells/well) were seeded in 96-well plates (TPP. Switzerland), cultured in DMEM containing 10% FCS (Lonza, Switzerland) with 13b or vehicle (DMSO), and formalin-fixed at days 0,1,2 and 3. To exclude the possibility that DMSO as vehicle affected the cell viability per se, final DMSO concentrations were the same for each concentration of 13b and the controls. Fixed cells were stained for 5 minutes with 50 ul/well of 0.3% Janus B Green dye (Acros Organics, Belgium) at room temperature with constant stirring followed by a de-staining step with water. Dye was eluted with 200 ul/well of 0.5 M HCl of hydrochloric acid and top-read measurements of absorbance were performed in a microplate reader (Sinergy HT, Biotek, USA) at 595 nm.

### Myography on Porcine Coronary Arteries

Myography on porcine coronary artery rings was performed as described in detail previously [29], [30]. In brief, left anterior descending arteries or posterior descending arteries were cut into rings (3 mm long) and rings were mounted onto a isometric force transducer (Pioden UF1, Graham Bell House, Canterbury, UK). The organ bath contained a 5 ml Krebs buffer maintained at 37°C and gassed with 95% O_2_/5% CO_2_. The composition of the Krebs buffer was as follows (in mM): NaCl 120, NaHCO_3_ 24.5, CaCl_2_ 2.4, KCl 4.7, MgSO_4_ 1.2, KH_2_PO_4_ 1 and glucose 5.6, pH 7.4. Each ring was passively stretched to an initial tension of 1 gr (10 mN). Changes in force were monitored and recorded on a computer using a Mac Lab System/8e program (AD Instruments Inc, Milford, Ma, USA), and digitalized at a sample rate of 0.5 sec.

To measure specifically endothelium-derived hyperpolarization-mediated vasorelaxation (EDH), the NO-synthase blocker, Nω-nitro-L-arginine (L-NNA, 300 µM), and the cyclooxygenase blocker, indomethacin (10 µM), were added to the buffer. Preparations were washed three times and were allowed to equilibrate for 40 min. Within this period, the incubation medium was renewed every 20 min. Compounds, alone or in combination, 13b (0.5 µM), SKA-31 (1 µM or 10 µM), 13b+SKA-31 (1 µM or 10 µM), or vehicle (DMSO) were tested as follows: First, rings were pre-incubated with one compounds or the combination of two for 5 min before the addition of serotonin (5-hydroxytryptamine, 5-HT, 1 µM, 1^st^ stimulation). Ten min later, bradykinin (1 µM) was added for another 10 min, followed by washout (over at least 10 min). This protocol was repeated (2^nd^ stimulation). Thereafter, we performed a 3rd round of pre-contraction and vasorelaxation, but using the vasospasmic thromboxane analogue U46619 (0.2 µM) as vasocontracting agent. At the end of the experiments, rings were allowed to contract maximally in a KCl (60 mM) buffer for 10 min followed by addition of the compounds. Finally, sodium nitroprusside (10 µM) was added to achieve maximal endothelium-independent vasorelaxation.

Stock solutions of 13b, SKA-31, and U46619 were made in DMSO at the day of the experimentation and added to the Krebs buffer in appropriate amounts to achieve the desired final concentration. DMSO as the vehicle had no vasoactive effect per se on the tissue. Other compounds were dissolved in Milli-Q water.

For data analysis, we determined absolute increases in force to 5-HT, U46619, or 60 mM KCl. Bradykinin-induced relaxations were determined as % change of pre-contraction to either vasocontracting compound and relative to the fully relaxed state (in the absence of the vasocontracting agent).

### Compounds and Chemicals

The trivanillic esters, 13a, 13b, and 13c were kind gifts of Dr. Delphine Lamoral-Theys, Laboratoire de Chimie Analytique, Toxicologie et Chimie Physique Appliquée, Université Libre de Bruxelles (ULB), and Dr. Robert Kiss, Laboratoire de Toxicologie, Institut de Pharmacie, ULB, Belgium, and were synthesized as described previously [22], [23]. Hydroxytyrosol was a kind gift of Dr. Jesús Osada, Department of Biochemistry and Molecular and Cellular Biology, Veterinary School, Health Research Institute of Aragon, CIBEROBN, Zaragoza, Spain. The other compounds were purchased from Sigma/Aldrich, Tocris, Alfa Aesar, or derived from in-house synthesis (TRAM-34 [5], SKA-31 [27]). Stock solutions (at 1 or 10 mM) of all compounds were prepared with dimethylsulfoxide (DMSO). The final DMSO concentration did not exceed 0.5% in single experiments testing one or more compounds.

### Statistics

Data are given as means ± SEM. Data sets were compared using one-way analysis of variance (ANOVA) and paired two-tailed Student’s T test if appropriate and as indicated in the legend text. P-values of <0.05 were considered statistically significant.

## Results

### Identification of Natural Product, Caffeic Acid, the NSAID, Flufenamic Acid, and the Synthetic Trivanillic Ester, 13b, as Novel Small Molecule Inhibitors of KCa3.1 Channels

**Figure 1:**
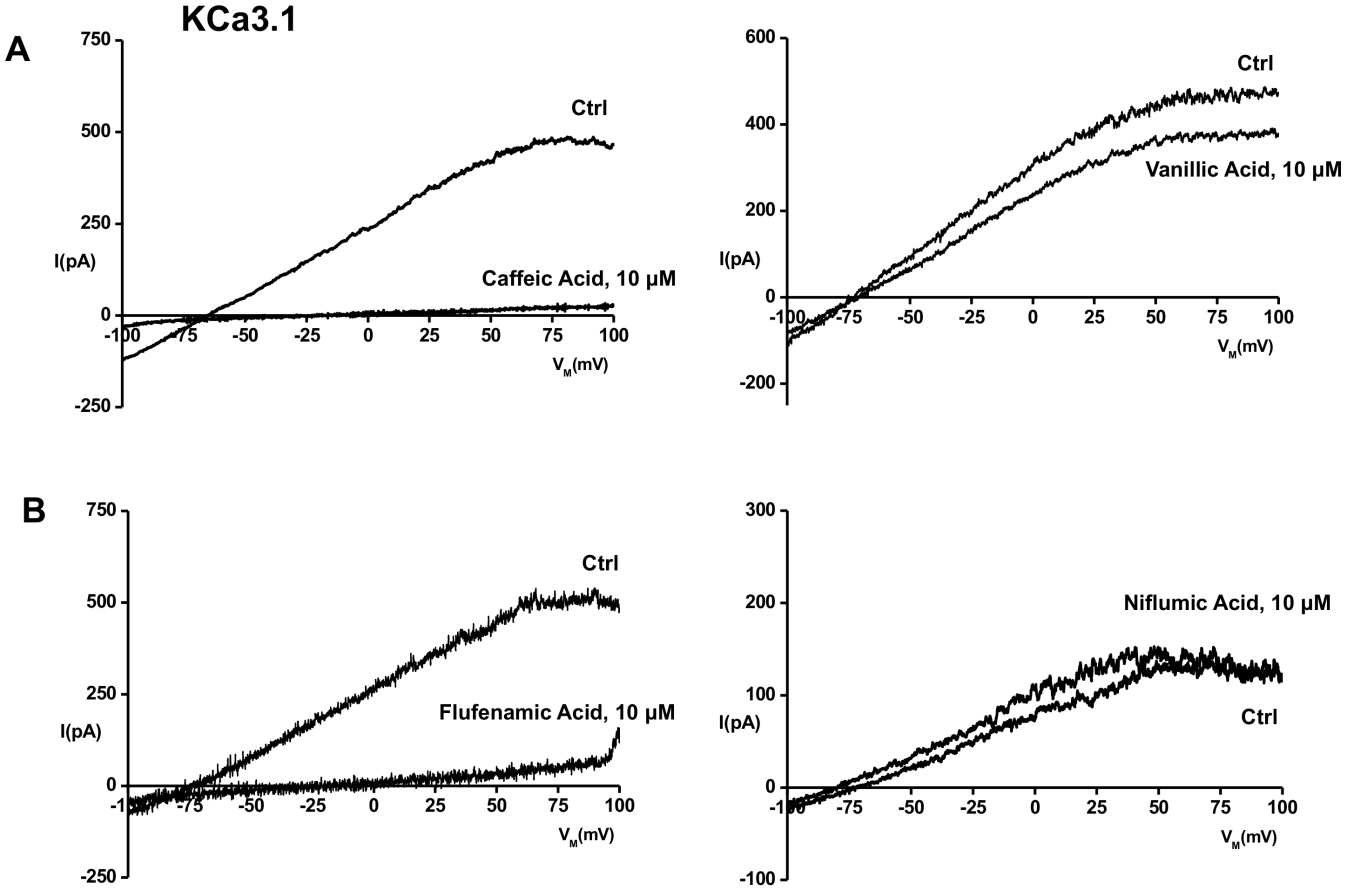
Pharmacological modulation of KCa3.1 channels by natural phenols and NSID. Original recordings of KCa3.1 whole-cell currents in 3T3 fibroblasts are shown. Currents were activated by infusion of 1 µM Ca^2+^_free_ via the patch-pipette and exhibited voltage-independence and inward-rectification typical for KCa3.1. A) On left: Complete inhibition of KCa3.1 channels by caffeic acid. On right: Weak inhibition by vanillic acid. B) On left: Complete inhibition of KCa3.1 channels by flufenamic acid. On right: The structurally similar niflumic acid had no blocking activity.

KCa3.1 channels in 3T3 fibroblasts [31] were activated by infusion of 1 µM Ca^2+^ and showed KCa3.1-typical voltage-independence and pronounced inward rectification at positive membrane potential similar to previous reports on human and rodent KCa3.1 [8], [32]. Our electrophysiological screening of 20 phenolic, benzoic, and polyphenolic natural products on KCa3.1 channels in 3T3 fibroblasts (as summarized in Table 1, for structures see Figure S1) revealed no (hydroxytyrosol, gallic acid, caffeic acid phenethyl ester, 1,2,3,4,6-pentagalloyl glucose), weak (vanillic acid, and ferulic acid, both with EC50s of >10 µM), or moderate blockade (caffeic acid, EC50 1.3±0.2 µM, Figure 1A; resveratrol, EC50 of ≈10 µM).

The synthetic benzoic acid derivatives, 3-fluoro-4-hydroxybenzoic acid and 4-amino-3-fluorobenzoic acid, had no appreciable blocking effects (Table 1). In contrast, the established KCa3.1 blocker TRAM-34 [5] completely inhibited the KCa3.1 current at 1 µM as expected (Table 1 at bottom). The NSAIDs had variable KCa3.1 blocking effects: Of the salicylates, 2-acetyloxybenzoic acid (Aspirin™) had no effect while mesalamine had weak blocking effects (EC50 26±8 µM). Of the fenamates, flufenamic acid was found to be a KCa3.1 inhibitor with moderate potency (EC50 1.6±0.1 µM, Figure 1B) while niflumic acid and mefenamic acid had no blocking effects at concentrations up to 50 µM. Modafinil, a synthetic biphenolic and analeptic drug that have been previously shown to block KCa3.1 by increasing cAMP-mediated phosphorylation [33], was found to be a weak direct channel inhibitor (EC50 of >1 µM, Table 1) in the present study. The 5-methyl-1-phenylpyridin-2-one, pirfenidone, a drug approved for the treatment of idiopathic lung fibrosis [34], had no effects on KCa3.1.

**Figure 2:**
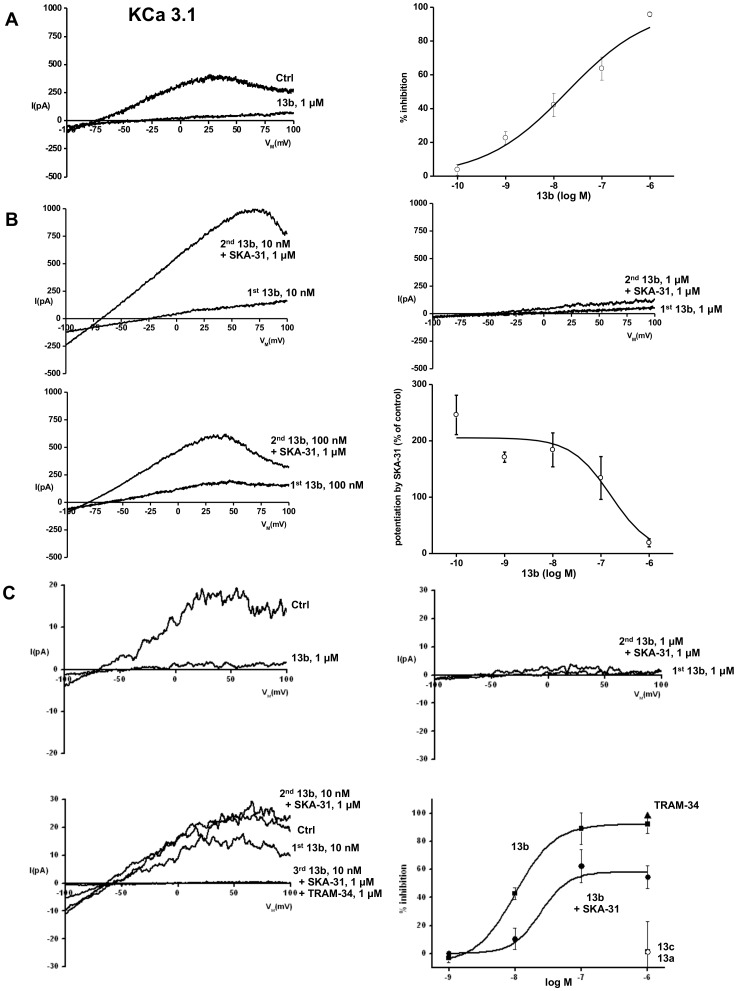
Inhibition of KCa3.1 channels by polyphenolic trivanillic ester, 13b. Original recordings of fibroblast KCa3.1 currents pre-activated by infusion of 1 µM Ca^2+^_free_ via the patch pipette. A) On left: Complete inhibition of KCa3.1 currents by 1 µM 13b. On right: Dose-response relationship for 13b. Fitting data points (representing means ± SEM, n = 4–9 each) gave an EC50 of 19 nM. B) Upper panel on left: Reversibility of KCa3.1 blockade with 10 nM 13b (applied 1^st^) by 1 µM SKA-31 (2^nd^). Lower panel on left: Moderate reversibility of KCa3.1 blockade with 100 nM 13b (applied 1^st^) by 1 µM SKA-31 (2^nd^). Upper panel on right: Poor reversibility of KCa3.1 blockade with 1 µM 13b (applied 1^st^) by 1 µM SKA-31 (2^nd^). Lower panel on right: Summary of data for reversibility. Data points are given as mean ± SEM (n = 4–8 each). C) Inhibition of hKCa3.1 currents by 13b in inside-out patches from hKCa3.1 overexpressing HEK293 cells. The hKCa3.1 currents were activated by excising the patch and exposure of the cytosolic face of the channel to the high K^+^ and 0.5 µM Ca^2+^_free_ containing bath solution. The pipette solution was contained physiological amounts of Na^+^ and K^+^. Upper panel on left: Inhibition of hKCa3.1 by 1 µM 13b. Lower panel on left: Half inhibition of KCa3.1 by 10 nM 13b (1^st^), full recovery after addition of 1 µM SKA-31 (2^nd^), and full blockade of the recovered current by 1 µM TRAM-34 (3^rd^). Upper panel on right: SKA-31 was not very effective at reversing the blockade caused by 1 µM 13b. Lower panel on right: Dose-response curves for the 13b blockade in inside-out patches (filled squares, n = 3–4) and recovery by 1 µM SKA-31 (filled circles, n = 3–4 each). Fitting of the data points (filled squares) gave an EC50 of 14 nM. TRAM-34 produced complete channel blockade of the recovered currents (open triangles, n = 2) while 1 µM 13c (open squares, n = 2) and 1 µM 13a (filled boxes, n = 2) had no or minor blocking effects.

Of the diversely substituted trivanillic esters (Table 1), 13b had considerable potency (Table 1 & Figure 2 A) as it blocked the channel at concentrations in the low nanomolar range (EC50 19±6 nM, Figure 2 A, on right). The Hill coefficient was close to 1, suggesting non-cooperative binding**.** The differently substituted analogues 13c and 13c had only weak to moderate blocking efficiency (Table 1) which could also be related to their even poorer solubility (13a, LogP 7.4; 13c, LogP 8.8 vs. 13b LogP 6.5) and possible precipitation in the physiological buffer used here. 13b also produced channel inhibition (4±1% of control, n = 5) with a Ca^2+^ concentration as high as 100 µM at the cytosolic face of the membrane and in the absence of the Ca^2+^-chelator EGTA, ruling out that the blocking effects of 13b were caused by interference (buffering) with cytosolic Ca^2+^ concentrations and Ca^2+^-sensitivity of the channel to higher Ca^2+^ concentrations (Figure S2).

### Reversibility of the Channel Blockade by the Positive Gating Modulator SKA-31

The channel blockade caused by submicromolar concentrations of 13b was reversed by 1 µM of the positive gating modulator of KCa3.1/KCa2 channels, SKA-31 [27] (see Table 1 for channel activation and Figure 2B, panels on left for reversibility of blockade). However, SKA-31 was substantially less effective at rescuing the current blocked by 1 µM 13b (Figure 2B, upper panel on right). The calculated EC50 of 13b to suppress the SKA-31-induced current was 161±121 nM. When the less potent inhibitors caffeic acid and flufenamic acid were used, 1 µM SKA-31 was able to recover the KCa3.1 current blocked by 10 µM flufenamic acid (6±1% of control, n = 3) to half the level of the initial current (53±20% of control). The current blocked by 10 µM caffeic acid (28±10% of control, n = 4) was likewise recovered by 1 µM SKA-31 (59±11% of control) but similar to flufenamic acid the current amplitude did not reach its initial amplitude. These dose-dependent antagonizing effects of 13b (and the weaker ones of flufenamic acid and caffeic acid) suggested an interaction of the inhibitor and the activator with the channel protein at the same or nearby binding site(s) with a higher affinity of 13b than for SKA-31, but we cannot rule out allosteric antagonism.

### Single Channel Experiments

In a series of inside-out experiments on the cloned human KCa3.1 channel overexpressed in HEK293 cells (Figure 2C), we tested whether the blocking effect of 13b was indeed due to a direct effect on the channel or possibly mediated through alteration of enzymatic channel regulation [35], [36], [37] or elicited by potential metabolites of 13b. These experiments revealed that 13b blocked the channels in the “isolated” inside-out patch with a similar EC50 of 14±3 nM and a Hill coefficient close to 1 (Figure 2 C, lower panel on right). Similar to the whole-cell experiments, SKA,-31 was also effective in antagonizing the blocking effects of nanomolar concentrations of 13b (Figure 2 C, lower panel on left) but could not recover currents blocked by 1 µM of 13b (Figure 2 C, upper panel on right). Again similar to the whole-cell experiments, the other derivatives, 13a and 13c, did not block the “isolated” channel in inside-out patches. TRAM-34 at 1 µM produced a complete block of channel currents in the absence of SKA-31 and of the currents recovered by SKA-31 (Figure 2C, lower panel on right and left).

### Selectivity of 13b

**Figure 3:**
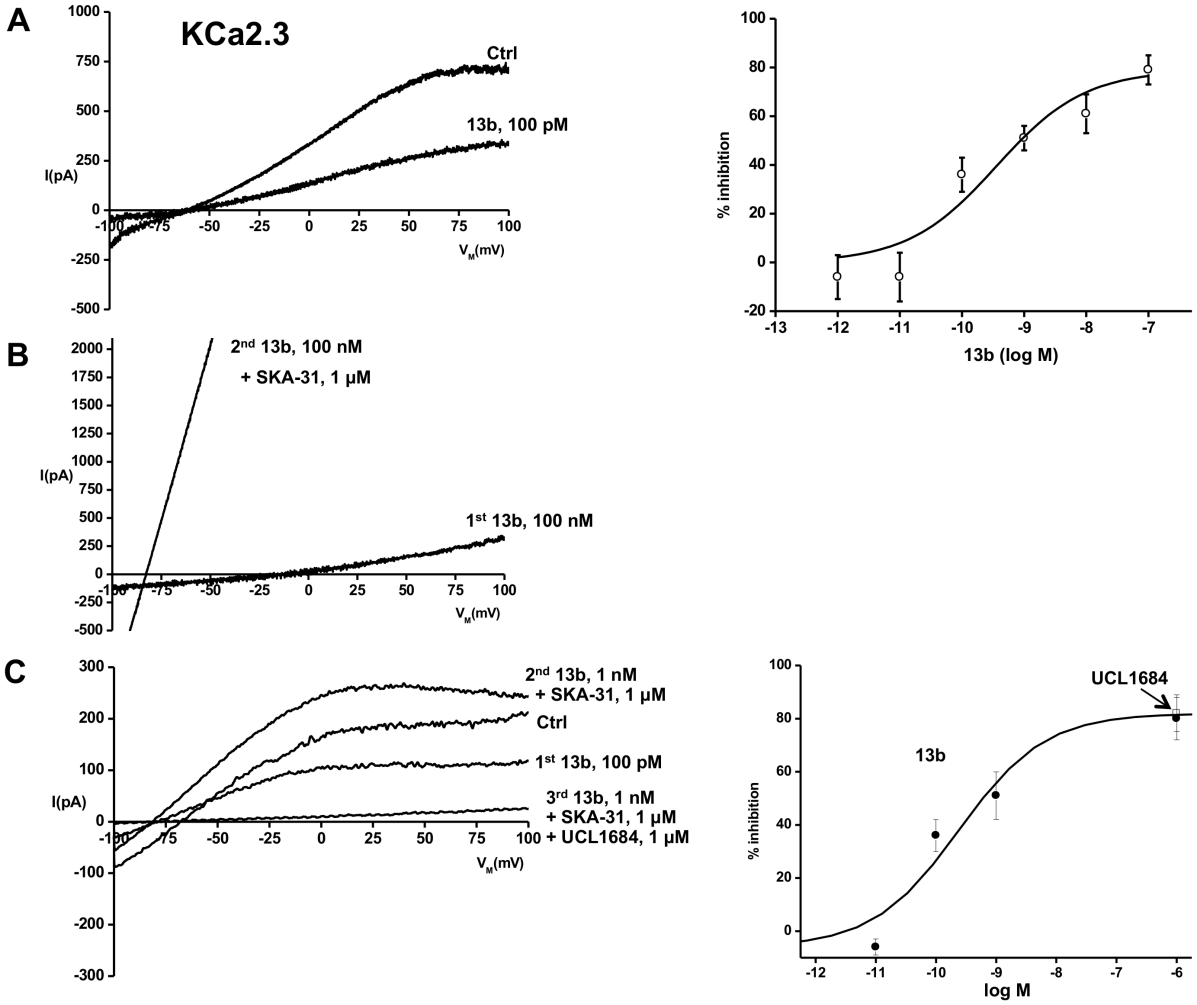
Inhibition of closely related hKCa2.3 by 13b. In fast whole-cell experiments, cloned hKCa2.3 were activated by infusion of 1 µM Ca^2+^_free_ via the patch-pipette. A) Panel on left: 13b at 100 pM inhibited KCa2.3 currents by approx. half. Panel on right: Dose-response relationship. Fitting of data points (representing means ± SEM, n = 3–9 each) gave an EC50 of 360 pM. B) Reversibility of complete channel blockade (at 100 nM 13b) by 1 µM SKA-31. C) Inhibition of hKCa2.3 by 13b in inside-out single-channel experiments. On left: original traces of inhibition by 100 pM 13b and SKA-31-induced recovery of currents blocked by 1 nM 13b. Full inhibition of the SKA-31-induced currents by UCL1684. On right: summary of data and dose-response curve. Data are given as means ± SEM, n = 2–5 each.

We next tested whether 13b would also affect the related KCa2 channels and indeed found that 13b blocked the human KCa2.3 in COS7 cells, although with a substantially lower EC50 of 360±173 pM (Figure 3A, upper panels). Like for KCa3.1, the Hill coefficient was close to 1, suggesting again non-cooperative binding of 13b**.** The rescue of the current by SKA-31 was similar to that observed in KCa3.1-expressing cells (Figure 3B).

As expected, the widely used selective KCa2 channel blocker UCL1684 [38], that binds to the outer vestibule of the channel, fully blocked (2±1% of control at 100 nM, n = 4) the hKCa2.3 current pre-activated by 1 µM SKA-31 (1157±485% of control, n = 4; Figure S3). In contrast to KCa3.1 channels, caffeic acid, flufenamic acid, 13a, and 13c had no blocking effects on KCa2.3 at micromolar concentrations (Table S1). Like for KCa3.1, we confirmed blocking efficacy of 13b in inside-out single channel experiments and determined a slightly lower EC50 of 241±129 pM and a Hill coefficient close to 1 (Figure 3 C). SKA-31 recovered the current blocked by 1 nM 13b (Figure 3 C, traces on left, summary of data and dose-response curve on right).

In contrast to KCa2 and KCa3.1, the phylogenetically distantly related large-conductance voltage-gated and non-calmodulin-regulated KCa1.1 in human U251 glioblastoma cells was not blocked by 13b (Table S1) while flufenamic acid at 10 µM was found to potentiate KCa1.1 by 2.7-fold similar to a previous report [39] and caffeic acid at 10 µM had no blocking or activating effects (Table S1). The cloned human voltage-gated K^+^ channel, hKv1.2, showed moderate sensitivity to 13b at 100 nM (19% inhibition) and was half-maximal block at 0.5 µM (EC50 0.55±0.01 µM, Figure S4 and Table S1), suggesting that 13b loses selectivity for KCa3.1/KCa2 channels in the micromolar range. Flufenamic acid and caffeic acid did not block hKv1.2 at 10 and 50 µM (Table S1). The other trivanillic ester 13a blocked hKv1.2 currents by 50% at 1 µM while 13c at 1 µM had no effect (Table S1). Cloned hKv1.3 channels were not inhibited by 13b at a concentration of 10 µM (Table S1). Also, cloned voltage-gated hERG channels were not blocked by either 13b or caffeic acid, while flufenamic acid caused a slight potentiation as also described previously [40] (Table S1). A native inward-rectifying K^+^ current (Kir) in U251 glioblastoma cells was not blocked by 13b at 1 µM (98±2% of control at −100 mV, n = 5), suggesting no blocking effects on this structurally different class of K^+^ channels.

### Cell Proliferation Assay

**Figure 4:**
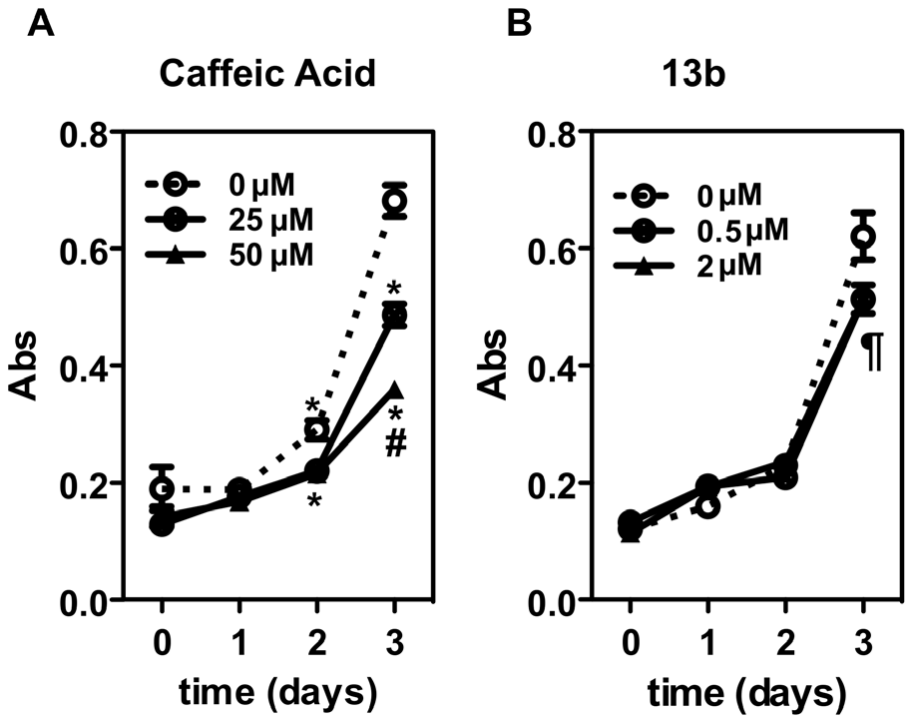
Inhibition of proliferation of 3T3 fibroblasts. 3T3-L1 fibroblasts were treated with increasing doses of the DMSO-solubilized phenolic compounds caffeic acid (A) and 13b (B) for 3 days. Experiments were repeated three times and data (absorption, ABS) were expressed as the means ± SEM of 8 replicates for each condition. Student’s T-test was used for statistical comparison of data sets at any given time point. For caffeic acid: *p<0.01 vs. control (vehicle), #p<0.001 vs. 25 µM; **¶**p<0.05 for 13b at 0.5 and 2 µM vs. control.

High functional expression of KCa3.1 has been proposed to promote cell proliferation in various tissues [21] and cell lines including fibroblasts [13], [14] and pharmacological inhibition of the channel has been shown to reduce cell proliferation [5], [11], [14], [19], [20]. To demonstrated utility of caffeic acid and 13b as KCa3.1 inhibitor in the present study, we evaluated the effects of the compounds on the proliferation of 3T3 fibroblast by a colorimetric assay. As shown in figure 4, cells proliferated in a time-dependent fashion regardless of the treatment. However, increasing doses of caffeic acid significantly slowed down the proliferation of 3T3 cells in a dose-dependent manner compared to vehicle-treated controls (−27% and −56% at 25 µM and 50 µM, respectively; Fig. 4A). Likewise, 13b inhibited cell proliferation at day 3 by 20%. However, we did not observe differences between the two doses (0.5 and 2 µM; −23 and –20%; Fig. 4B).

### Blockade of Native Porcine Endothelial and Vasoactive Properties of 13b

KCa3.1 and KCa2.3 are key players in the endothelium-dependent control of arterial tone by producing endothelial hyperpolarization and thereby endothelium-dependent vasorelaxation resistant to inhibition of NO- and prostacyclin synthesis [21]. As a further proof of functionality and utility of 13b as a potent KCa3.1/KCa2.3 inhibitor in a more complex physiological setting, we determined sensitivity of KCa3.1 and KCa2.3 functions to 13b in native porcine coronary artery endothelial cells and tested by isometric myography on porcine coronary artery rings whether 13b altered relaxation to bradykinin alone and to bradykinin in combination with SKA-31. In addition, we tested whether 13b modified contractions to serotonin (5-HT) and the vasospasmic thromboxane analogue U46619. For this, we chose a concentration of 0.5 µM of 13b which was more than 10-fold above the EC50 for KCa3.1 and KCa2.3 channels but had little blocking effects on hKv1.2 channels possibly expressed in the smooth muscle of these coronary arteries.

**Figure 5:**
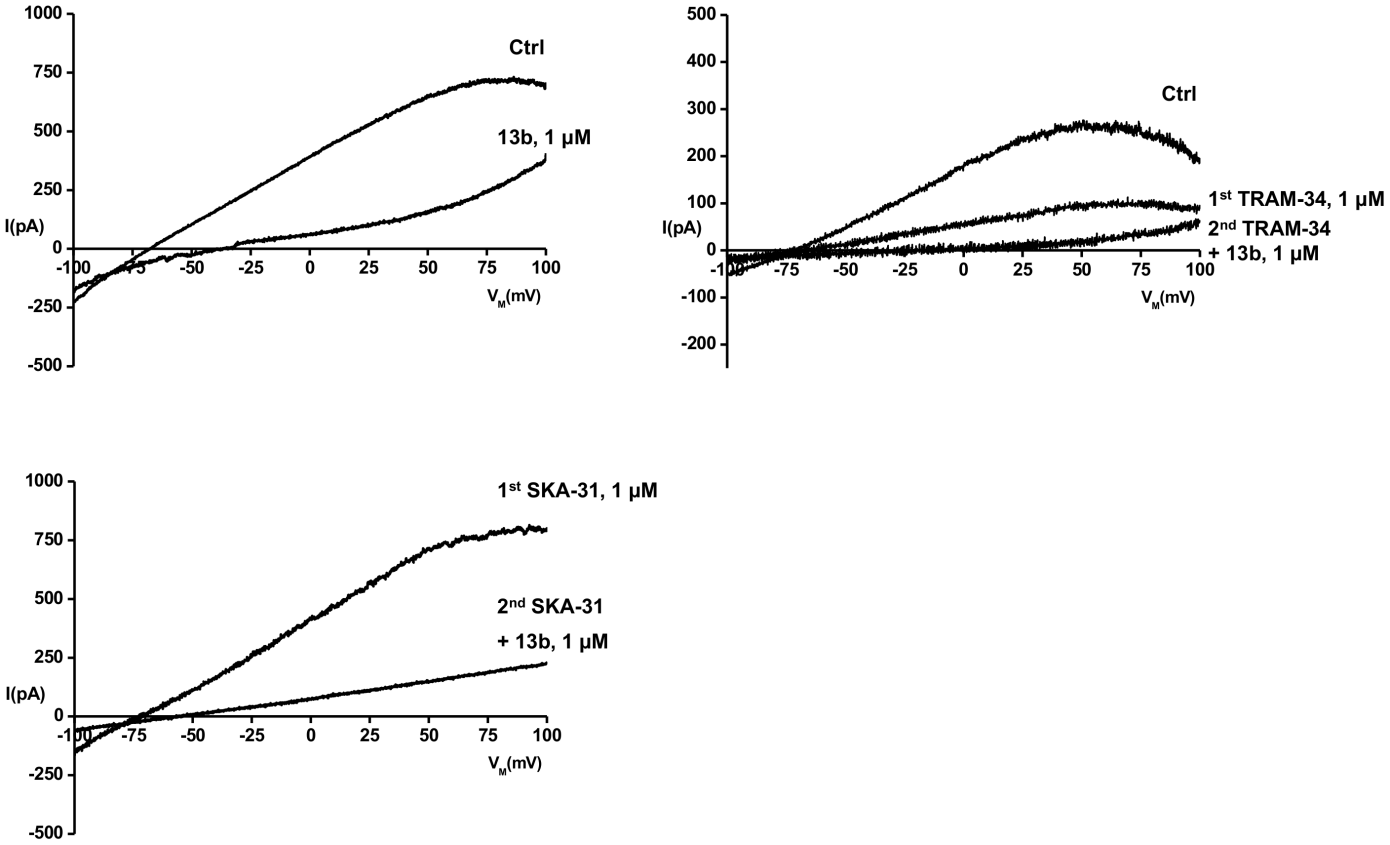
Inhibition of KCa channels by 13b and TRAM-34 in freshly isolated porcine coronary artery endothelial cells. Representative whole-cell current recordings are shown. Upper panel on left: 13b-blockade of KCa currents (activated by infusion of 1 µM Ca^2+^_free_ via the patch-pipette; cells, n = 3). Upper panel on right: Blockade of KCa3.1 current by 1 µM TRAM-34 and blockade of the residual current (KCa2.3) by 13b (n = 4). Lower panel on left: Blockade of SKA-31-activated currents by 1 µM 13b (n = 1).

Using whole-cell patch-clamp experiments we first confirmed that PCAEC like other species expressed mixed KCa3.1 and KCa2.3 currents (Figure 5). These currents with a mean amplitude of 30±8 pA/pF (n = 9) were almost completely blocked by 1 µM 13b (14±7% of control, n = 3). The KCa3.1 blocker TRAM-34 blocked half of the total KCa current at 1 µM (58±4% of control, n = 4) and the remaining current mediated by KCa2.3 was blocked by 1 µM 13b (9±4% of control, n = 4). A current potentiated by 1 µM SKA-31 was almost fully blocked by 1 µM 13b. Flufenamic acid at 10 µM blocked the mixed KCa3.1/KCa2.3 to 25±3% of control (n = 2). Flufenamic acid and caffeic acid, both at 50 µM (n = 1) blocked the currents almost completely to 3 and 6% of control, respectively.

Myography data (means, SEM, and *n*, *P* values from statistical calculations) were summarized in the tables 2, 3, 4 and showed that 13b at 0.5 µM did not produce contractions in its own right or interfered with endothelium-independent contractions to 60 mM K^+^ or with the endothelium-independent relaxations to the NO-donor sodium nitroprusside (Tables S2 and S3). Likewise, the KCa3.1/KCa2.3 activator SKA-31 at 1 or 10 µM did not produce contractions or relaxations in its own right or interfered with contractions to 60 mM K^+^ or relaxations to sodium nitroprusside (Tables S2 and S3). In contrast, 0.5 µM 13b augmented the amplitude of contraction induced by 1 µM 5-HT by +74% when compared to vehicle (DMSO)-treated rings (P<0.01; Table 2). SKA-31 at 1 or 10 µM as well as SKA-31 in combination with 0.5 µM 13b did not modify contraction to 5-HT, with the only exception of reduced contractions in the presence of 10 µM SKA-31 plus 0.5 µM 13b (−58%, P<0.01 vs. vehicle; Table 2). After wash out (20 min), a repetition of the protocol gave similar results (2^nd^ stimulation in Table 2), i.e. a stronger contraction with 13b (+90%, P<0.01 vs. vehicle), and weaker contractions in the presence of 10 µM SKA-31 (−44%, P<0.05 vs. vehicle) or 10 µM SKA-31 plus 0.5 µM 13b (−44%, P<0.05 vs. vehicle).

**Table 2:** 13b and SKA-31 modulate 5-HT-induced contractions in porcine coronary artery.

Compound(s)	1^st^ stimulation			2^nd^ stimulation		
	n	Δg	*P* vs. Ve	n	Δg	*P* vs. Ve
Vehicle (Ve)	7	0.4±0.1		7	0.4±0.1	
13b 0.5 µM	8	0.7±0.1	<0.5	7	0.7±0.1	<0.01
SKA-31 1 µM	4	0.4±0.1	n.s.	4	0.5±0.1	n.s.
SKA-31 10 µM	5	0.4±0.1	n.s.	5	0.23±0.02	<0.5
13b 0.5 µM+SKA-31 1 µM	4	0.4±0.1	n.s.	4	0.6±0.1	n.s.
13b 0.5 µM+SKA-31 10 µM	5	0.16±0.02	<0.01	5	0.22±0.03	<0.5

Data are given as mean ± SEM; n.s. not significant.

**Table 3:** 13b and SKA-31 modulate U46619-induced contractions in porcine coronary artery.

Compound(s)	n	Δg	*P* vs. Ve
Vehicle (Ve)	8	2.2±0.2	
13b 0.5 µM	7	2.8±0.2	<0.5
SKA-31 1 µM	4	1.9±0.5	n.s.
SKA-31 10 µM	4	1.7±0.2	n.s.
13b 0.5 µM+SKA-31 1 µM	4	2.3±0.4	n.s.
13b 0.5 µM+SKA-31 10 µM	4	1.9±0.2	n.s.

Data are given as mean ± SEM; n.s. not significant.

**Table 4:** Modulation of bradykinin-induced relaxation in porcine coronary artery by 13b and SKA-31.

Compound(s)		5-HT-precontraction			U46619-precontraction	
	n	% relaxation	*P* vs. Ve	n	% relaxation	*P* vs. Ve
Vehicle (Ve)	6	31±5		7	−11±2	
13b 0.5 µM	7	24±2	n.s.	7	−1±1	<0.001
SKA-31 1 µM	4	65±6	<0.01	4	5±1	<0.001
SKA-31 10 µM	5	47±8	<0.05	5	10±2	<0.001
13b 0.5 µM+SKA-31 1 µM	3	38±8	n.s.	4	2±1	<0.001
13b 0.5 µM+SKA-31 10 µM	5	69±4	<0.001	5	8±2	<0.001

Data are given as mean ± SEM; n.s. not significant.

As shown in table 3, the vasospasmic thromboxane analogue U46619 (0.2 µM) produced substantially stronger contractions (amplitude of force: U46619, 2.2 g vs. 5-HT, 0.35 g). These contractions were further enhanced (+29%) by 13b (P<0.05 vs. vehicle). SKA-31 at 1 µM and 10 µM did not significantly modify these contractions but prevented the stronger contractions caused by 13b (−5%, n.s., and −14%, n.s. vs. vehicle, respectively).

As shown in table 4, bradykinin at 1 µM produced a 30% relaxation of 5-HT-precontracted and vehicle-treated rings (1^st^ 5-HT contraction). This relaxation was not significantly reduced by 13b (−27%, n.s. vs. vehicle). In contrast, SKA-31 at 1 and 10 µM augmented the relaxation (+110% and +52%, respectively; P<0.01 vs. vehicle) and 13b at 0.5 µM was efficient to largely prevent the potentiation of the bradykinin response caused by 1 µM SKA-31 (+23%, n.s. vs. vehicle) but not by 10 µM (+123%; P<0.001 vs. vehicle). This finding was in line with the notion that high concentrations of SKA-31 antagonized the 13b blocking effects on KCa3.1 and KCa2.3 channels as observed in the electrophysiological experiments.

In experiments on U46619-precontracted rings, bradykinin failed to relax these strongly contracted rings, rather the rings continued contracting (+10% contraction in the presence of the vehicle). The rings also continued contracting in the presence of 0.5 µM 13b albeit to some lesser degree (+1% contraction; P<0.01 vs. vehicle). In contrast, combined stimulation with bradykinin and SKA-31 at 1 and 10 µM elicited small but appreciably relaxations (5% and 10% relaxation; both P<0.01 vs. vehicle) of these U46619-precontracted rings. 13b prevented by trend the relaxation produced by the combination of bradykinin plus 1 µM SKA-31 (2% relaxation, n.s. vs. bradykinin plus 1 µM SKA-31 alone), but not the relaxation produced by bradykinin plus 10 µM SKA-31 (8% relaxation, n.s.). The latter findings were again in line with the antagonizing effects of high concentrations of SKA-31 on 13b-induced channel inhibition. Together, the myography revealed that 13b was functionally active as a KCa3.1/KCa2.3 inhibitor in an artery by promoting vasoconstraction and counteracting endothelium-dependent vasorelaxation caused by pharmacological KCa3.1/KCa2.3 activation.

## Discussion

The main outcome of our study was: 1) the natural phenol caffeic acid and the NSAID, flufenamic acid, were found to be moderately potent KCa3.1 inhibitors with EC50s in the lower micromolar range. 2) The synthetic tri-fluoro trivanillic ester, 13b, was identified as a potent KCa3.1 inhibitor with a low EC50 of 19 nM. Moreover, 13b acted as a novel type of negative gating modulator of KCa3.1 channels as evidenced by the antagonizing capabilities of the positive gating modulator SKA-31. 3) 13b blocked closely related KCa2.3 channels with even higher potency (EC50 360 pM). 4) 13b was found to be non-toxic in cultured 3T3 fibroblasts and reduced proliferation at submicromolar concentration by 20%. 5) 13b blocked native KCa2.3/KCa3.1 channels in porcine endothelium and augmented contractions of porcine coronary arteries induced by 5-HT and or the vasospasmic thromboxane analogue U46619. 13b reduced the endothelium-dependent relaxation to combined stimulation with bradykinin and SKA-31. Together these findings identify 13b as new and potent pan-inhibitor of KCa2/KCa3.1 channels.

Natural phenolic or benzoic phytochemicals, although known to exert multiple pharmacological effects, have not been previously described to block KCa3.1/KCa2.3 channels. Our screening identified caffeic acid and resveratrol as the first natural phenolic phytochemicals with a remarkable KCa3.1-blocking efficacy as indicated by EC50s in the lower micromolar range (1–10 µM). Concerning the benzoic NSAIDs, flufenamic acid has previously been shown to block calcium-activated chloride channels as well as non-selective cation channels, with reported EC50s of approx. 30 and 5 µM, while an inhibitory effect on KCa3.1 has not been described so far. Here, we found that flufenamic acid was a moderately potent KCa3.1 inhibitor with an EC50 of 1.6 µM. From the pharmacological perspective, it could thus be tempting to speculate that inhibitory actions of either caffeic acid, resveratrol, or flufenamic acid on KCa3.1 channels were part of the mechanisms by which caffeic acid and flufenamic acid produced anti-inflammatory effects and altered carcinogenesis as described previously [41], [42]. Notwithstanding, the potencies of caffeic acid, resveratrol, and flufenamic acid as KCa3.1-inhibitors were moderate, but still may further advocate caffeic acid and resveratrol -as natural food additive-, or caffeic acid containing vegetable oils, like extra-virgin olive oil and argan oil, and the classical NSAID flufenamic acid for e.g. adjuvant immune suppressive or cytostatic treatments or for topical applications for inflammatory skin diseases.

The main outcome of our small scale screening study was the identification of the 3-fluoro trivanillic acid ester 13b as a nanomolar inhibitor of KCa3.1 channels, and, intriguingly, picomolar inhibitor of KCa2.3 channels. At present, this makes 13b the most potent known small molecule inhibitor of KCa2.3 whereas its potency on KCa3.1 was similar to the known and structurally unrelated blockers ICA-17043 (Senicapoc), TRAM-34, and NS6180 [43]. Interestingly, the meta-substitution of the fluoride atom of the phenol moiety by a methoxy group as in 13a or by a chloride atom as in 13c, virtually abolished efficacy of these analogues, suggesting that the fluoride is crucial for interaction with the channels.

While 13b did not discriminate very well between small-conductance KCa channels (KCa2) and the intermediate-conductance channel (KCa3.1), the distantly related voltage-gated large-conductance KCa channel KCa1.1 with another type of calcium-sensitivity (non-calmodulin-conferred) was not blocked by 13b. Concerning voltage-gated K^+^ channels, 13b had only moderate but still appreciable blocking effects on hKv1.2 channels (EC50 0.55 µM) while it did not block hKv1.3 channels and hERG channels. This selectivity profile indicated substantial selectivity for KCa2/KCa3.1 channels over voltage-gated K^+^ channels.

Regarding the potential binding site/interaction site of 13b, we suggest here that it might be located in the vicinity of the site at which the positive gating-modulator SKA-31 is acting because SKA-31 was capable to functionally antagonize 13b in our patch-clamp experiments. However, there is of course also the possibility that the antagonism is not direct but instead allosteric as between the negative gating modulator NS8583 and the positive gating modulator NS309 on KCa2.3 channels [44]. Unlike 13b, the classical KCa3.1 blocker TRAM-34, which is binding to a putative site below the selectivity filter of KCa3.1 [45] did not interfere with the positive gating modulation of SKA-31, but fully blocked currents activated by micromolar concentration of SKA-31 as well as 13b-pre-blocked/SKA-31-recovered currents. These different blocking properties of 13b and of TRAM-34 and the lack of interaction of both TRAM-34 and 13b argued in favor of a different binding site and molecular mechanism by which 13b causes channel blockade. Together, these data suggested that 13b could represent the first example of a new type of negative gating modulator for KCa3.1 channels. Interestingly, another negative gating modulator, NS8593 has recently been described for KCa2 channels [44], which interacts with the inner pore of KCa2 channels close to the internal gate at a same site at which TRAM-34 causes inhibition of KCa3.1. However, based on the much larger molecular size of 13b we believe it rather unlikely that 13b acts as a negative gating modulator by entering the narrow inner pore of KCa2/3 channels in the same way as TRAM-34 and NS8593. Another fact that clearly distinguishes the fluoro-trivanillic ester 13b from NS8593 is its lack of calcium-dependence. While NS8593 becomes less potent at higher internal Ca^2+^ concentrations, 13b can still fully block KCa2/3 channels even in the presence of 100 µM of free internal Ca^2+^.

Regarding the binding sites of the positive gating modulators of KCa3.1 and KCa2 channels, the positive gating modulators EBIO and NS309 (structurally related to SKA-31) were recently shown to bind in a pocket within the space between calmodulin and the C-terminal calmodulin-binding domain (CAMBD) as concluded from docking experiments using the recently solved structure of a co-crystal of CAM and the C-terminus of KCa2.2 into which EBIO had been soacked [46]. Assuming that SKA-31 uses the same binding pocket in KCa3.1 and KCa2.3 channels, we suggest that 13b might be binding in or near the same pocket where it could be displaced by SKA-31. However, we can of course not completely exclude allosteric effects mediated through interactions of 13b and SKA-31 with non-overlapping sites.

The second major aim of the study was to demonstrate the utility of this new and highly efficient inhibitor for studying cellular and physiological processes to which KCa3.1 and/or KCa2.3 channels have been proposed to contribute.

Treatment of 3T3 fibroblasts with 13b at a submicromolar concentration of 0.5 µM and at 2 µM moderately reduced cell proliferation (-20%), similar to that what had been previously observed in another murine renal fibroblast cell line using TRAM-34 as a KCa3.1 blocker [14]. Interestingly, caffeic acid had even more potent antiproliferative effects on 3T3 fibroblast although these were achieved at 50 and 100 times higher (micromolar) concentrations.

In the vascular endothelium, KCa3.1 and KCa2.3 channels are key players in the initiation of endothelium-derived hyperpolarization(EDH)-mediated arterial dilations [21]. Moreover, KCa3.1 activation has been suggested to be more important for EDH dilation following the stimulation of G-protein-coupled receptors while KCa2.3 channel function was reported to support the tonic vasodilating influence of the endothelium and shear stress-induced EDH dilation, thus assigning distinct functional roles to the channels for different endothelial vasodilator functions [47]. Considering 13b as an endothelial KCa3.1/KCa2.3 inhibitor, we expected that 13b would be vasoactive by influencing these endothelial functions. Indeed, our myography on porcine coronary arteries revealed that 13b augmented the contractile responses to 5-HT and to the vasospamic U46619 at a submicromolar concentration (0.5 µM). From the physiological perspective, these results suggested that the inhibition of endothelial KCa3.1 and KCa2.3 in porcine coronary arteries abolished a tonic (KCa2.3) and/or agonist-induced (KCa3.1) endothelium-derived negative feedback on 5-HT or U46619 induced contractions.

In keeping with the notion that SKA-31 antagonized 13b actions on the channels in patch-clamp experiments, we also expected SKA-31 antagonism of the pro-contractile effects of 13b. In fact, SKA-31 at the higher concentration of 10 µM fully antagonized the pro-contractile effects of 13b on 5-HT-induced contractions and even reduced the contractions elicited by 5-HT alone. In addition, SKA-31 also antagonized the pro-contractile effects of 13b on the strong contractions to U46619. Thus, these findings confirmed our idea that SKA-31 was capable of reversing the 13b-blockade of the channels and of promoting vasorelaxation in the presence of a vasocontracting neurotransmitter or a vasospamic agent.

Concerning endothelium-derived hyperpolarization-induced relaxation caused by G-protein-coupled receptor stimulation (here of bradykinin receptors), 13b had no significant effect on bradykinin–induced relaxation of 5-HT pre-contracted vessels, suggesting that both channels were not required for this type of relaxation in porcine coronary arteries. In contrast, SKA-31 as an endothelial KCa3.1/KCa2.3 activator greatly augmented the relaxation caused by bradykinin. 13b prevented this SKA-31-mediated potentiation of the bradykinin-induced relaxation at 1 µM SKA-31, indicating that 13b was capable to antagonize the vasorelaxation to combined stimulation with bradykinin by blocking the -under these conditions substantially active- endothelial KCa3.1/KCa2.3 channels. Because of the strong antagonizing effects of high doses of SKA-31 on the 13b blockade, 13b was predictably unable to reduce the larger bradykinin-induced relaxations at the higher 10 µM concentration of SKA-31. Concerning the U46619-precontracted vessels, bradykinin effectively produced relaxations of small amplitude only in the presence of SKA-31, suggesting that the endothelium in the absence of NO and prostacyclin synthesis was hardly able to counteract the strong tone produced by the vasospasmic U46619. 13b antagonized these weak relaxation responses only by trend at the lower concentration of SKA-31 while it had clearly no effect at the higher concentrations of SKA-31, thus supporting the idea that 13b did not modulate bradykinin responses but antagonizes effects caused by SKA-31.

13b did not alter contractions in response to high K^+^ concentrations and the relaxation to the NO donor sodium nitro prusside indicating that the compound did not interfere with calcium-dependent contractility of the smooth muscle and endothelium-independent relaxation and has therefore no gross unspecific effects on smooth muscle functions.

In sum, the myography experiments revealed that 13b was a vasoactive KCa3.1/KCa2.3 inhibitor and acted by augmenting vasocontraction to agonists and impaired EDH-vasorelaxation to combined bradykinin stimulation and pharmacological opening of endothelial KCa3.1/KCa2.3.

KCa3.1/KCa2.3 channels are key players in the proliferation of some cell types and cancer cell lines as well as in endothelial function [15], [21]. As a structurally novel, potent, and pan-KCa3.1/KCa2 channel active inhibitor 13b constitutes a new tool compound to further study the physiological and pathophysiological roles of these channels and might also have therapeutic utility in disease states, like chronic inflammation and autoimmune disease, atherosclerosis, fibrosis, cancer, and hypotension to which KCa3.1/KCa2 channel up-regulation or activation contribute.

## Supporting Information

Figure S1 Structures of compounds.
(TIF)

Figure S2 Inhibition of SKA-31-potentiated KCa3.1 current by 1 µM 13b in presence of a high Ca^2+^-concentration of 100 µM at the cytosolic face and in the absence of the Ca^2+^-chelator EGTA. The recording is representative of 5 experiments.
(TIFF)

Figure S3 Representative traces illustrating the inhibition of hKCa2.3 (pre-activated by 1 µM SKA-31) by UCL1684.
(TIFF)

Figure S4 13b blocked the activity of hKv1.2 overexpressed in HEK293 cells. The graph shows a dose-response curve and data points represent means ± SEM; n = 3–5 each). Fitting of the data revealed an EC50 of 0.55±0.01 µM.
(TIFF)

Table S1 Effects of caffeic acid, flufenamic acid, and trivanillic esters on other K^+^ channels.
(PDF)

Table S2 13b and SKA-31 did not modulate contractions to 60 mM K^+^ in porcine coronary artery.
(PDF)

Table S3 13b and SKA-31 did not modulate endothelium-independent relaxation to the NO donor, SNP, in porcine coronary artery pre-contracted by 60 mM K^+^.
(PDF)
