# Supplementary figures and images for: Novel Phenolic Inhibitors of Small/Intermediate-Conductance Ca2+-Activated K+ Channels, KCa3.1 and KCa2.3
Source: PLoS One. 2013 Mar 14;8(3):e58614. doi: 10.1371/journal.pone.0058614 (PMC3597730; doi:10.1371/journal.pone.0058614)

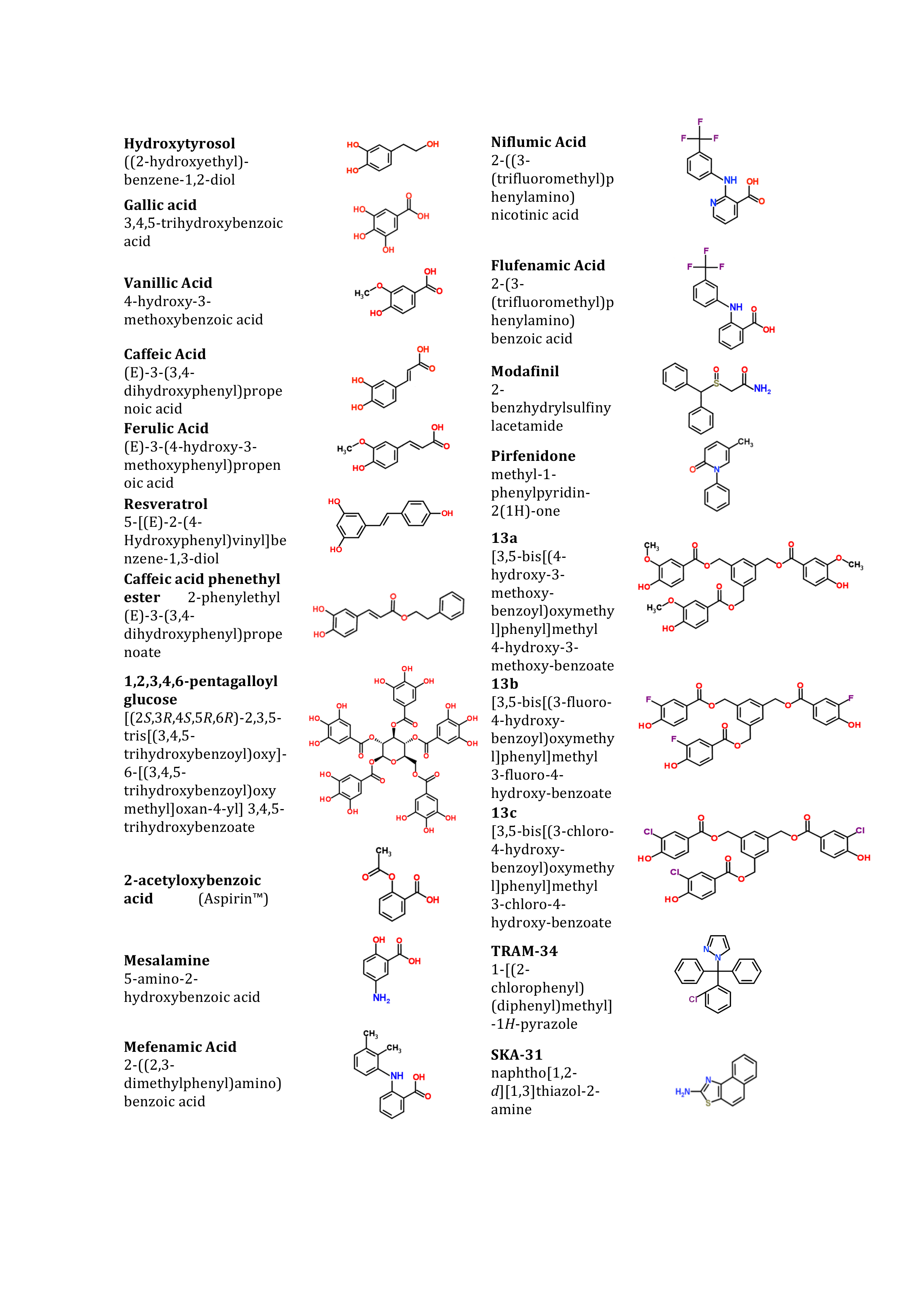

Supplement: Figure S1 — Structures of compounds. (TIF) [file pone.0058614.s001.tif]

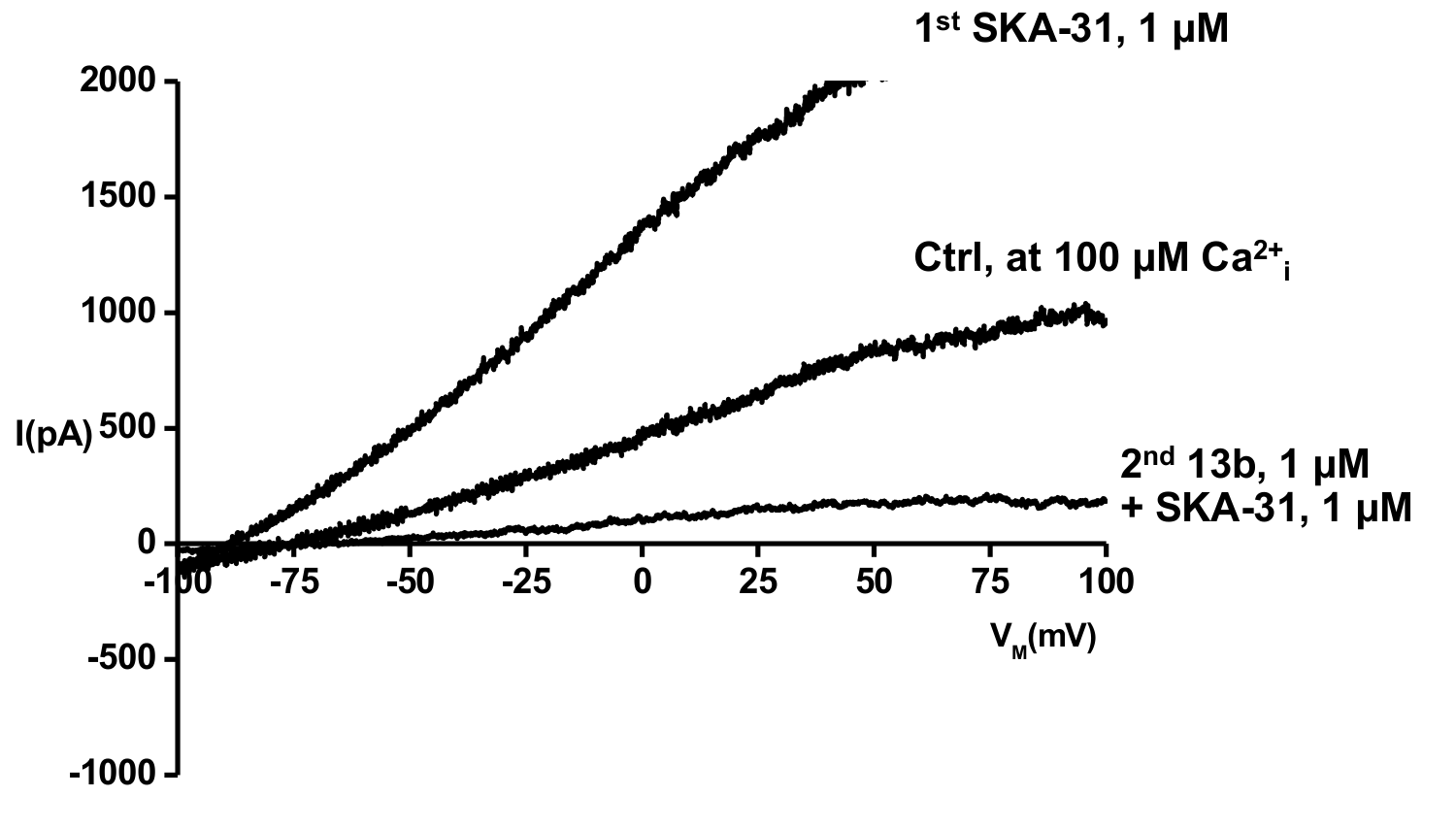

Supplement: Figure S2 — Inhibition of SKA-31-potentiated KCa3.1 current by 1 µM 13b in presence of a high Ca2+-concentration of 100 µM at the cytosolic face and in the absence of the Ca2+-chelator EGTA. The recording is representative of 5 experiments. (TIFF) [file pone.0058614.s002.tiff]

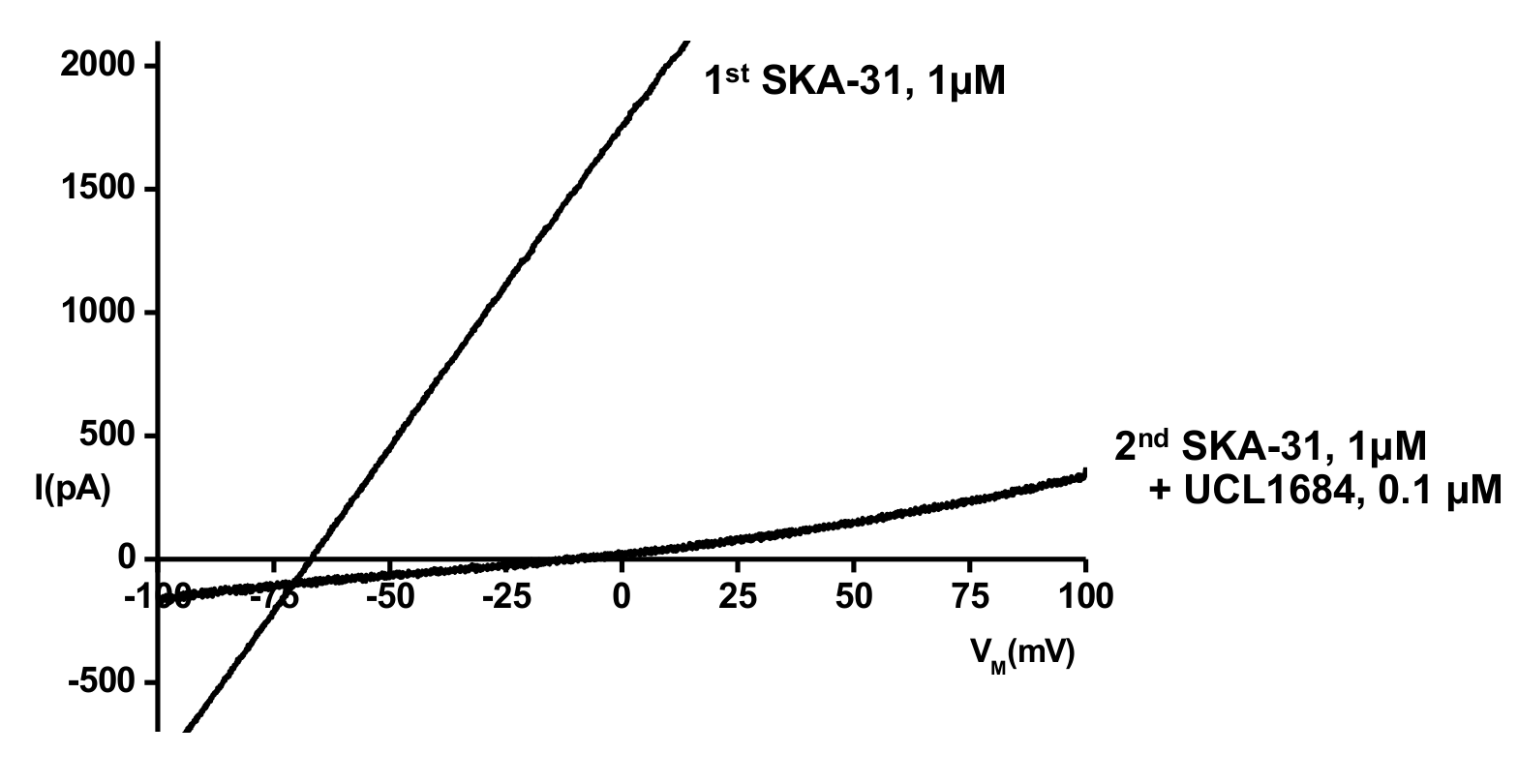

Supplement: Figure S3 — Representative traces illustrating the inhibition of hKCa2.3 (pre-activated by 1 µM SKA-31) by UCL1684. (TIFF) [file pone.0058614.s003.tiff]

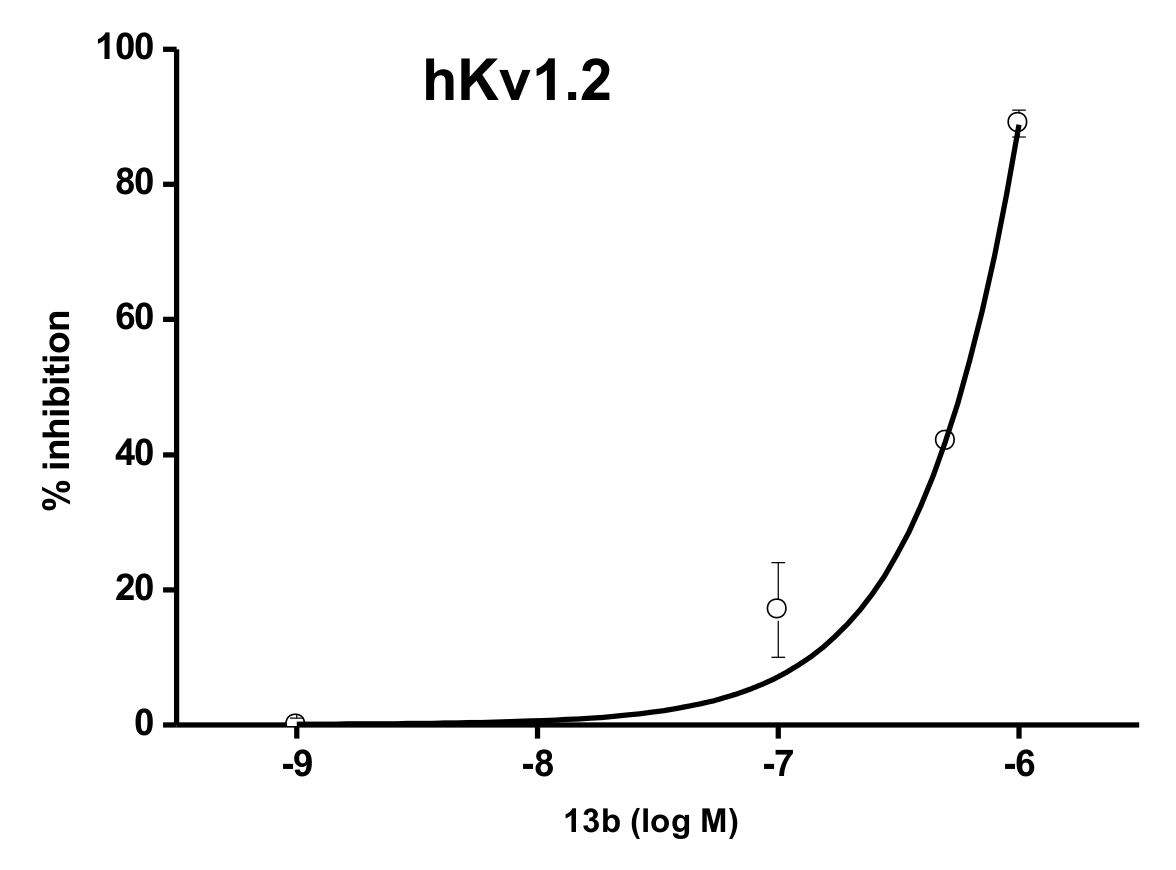

Supplement: Figure S4 — 13b blocked the activity of hKv1.2 overexpressed in HEK293 cells. The graph shows a dose-response curve and data points represent means ± SEM; n = 3–5 each). Fitting of the data revealed an EC50 of 0.55±0.01 µM. (TIFF) [file pone.0058614.s004.tiff]
