# Supplementary material for: Novel Phenolic Inhibitors of Small/Intermediate-Conductance Ca2+-Activated K+ Channels, KCa3.1 and KCa2.3
Source: PLoS One. 2013 Mar 14;8(3):e58614. doi: 10.1371/journal.pone.0058614 (PMC3597730; doi:10.1371/journal.pone.0058614)
Supplement: Table S1 — Effects of caffeic acid, flufenamic acid, and trivanillic esters on other K+ channels. (PDF) [file pone.0058614.s005.pdf]

Table S1: Effects of caffeic acid, flufenamic acid, and trivanillic esters on other K^+ channels.

Compound	% of control at		
	1 µM	10 µM	50 µM
**hKCa2.3**			
**Caffeic Acid**	NT	108 ± 7	97 ± 1
**Flufenamic Acid**	NT	104 ± 5	100 ± 2
**13a**	98 ± 1	NT	NT
**13b**	4 ± 2	NT	NT
**13c**	92 ± 4	NT	NT
**hKCa1.1**			
**Caffeic Acid**	NT	103 ± 14	NT
**Flufenamic Acid**	NT	270 ± 106 (activation)	NT
**13b**	103 ± 3	NT	NT
**hKv1.2**			
**Caffeic Acid**	NT	100 ± 2	97 ± 1
**Flufenamic Acid**	NT	106 ± 5	100 ± 2
**13a**	46 ± 3	NT	NT
**13b**	11 ± 2	NT	NT
**13c**	104 ± 3	NT	NT
**hKv1.3**			
**13b**	96.4 ± 0.2	NT	NT
**hERG**			
**Caffeic Acid**	NT	97 ± 6	NT
**Flufenamic Acid**	NT	116 ± 15	NT
**13b**	94 ± 3	NT	NT

NT, not tested; data are given as mean ± SEM, n ≥ 3
