# Supplementary material for: Novel Phenolic Inhibitors of Small/Intermediate-Conductance Ca2+-Activated K+ Channels, KCa3.1 and KCa2.3
Source: PLoS One. 2013 Mar 14;8(3):e58614. doi: 10.1371/journal.pone.0058614 (PMC3597730; doi:10.1371/journal.pone.0058614)
Supplement: Table S2 — 13b and SKA-31 did not modulate contractions to 60 mM K+ in porcine coronary artery. (PDF) [file pone.0058614.s006.pdf]

Table S2: 13b and SKA-31 did not modulate contractions to 60 mM K^+ in porcine coronary artery

Compound(s)	n	Δg	*P* vs. Ve
Vehicle (Ve)	7	2.3±0.3	
13b 0.5 µM	8	3.1±0.3	n.s.
SKA-31 1 µM	4	3.2±0.5	n.s.
SKA-31 10 µM	4	2.5±0.4	n.s.
13b 0.5 µM + SKA-31 1 µM	4	2.1±0.3	n.s.
13b 0.5 µM + SKA-31 10 µM	4	2.5±0.3	n.s.

Data are given as mean ± SEM; n.s. not significant.
