# Supplementary material for: Novel Phenolic Inhibitors of Small/Intermediate-Conductance Ca2+-Activated K+ Channels, KCa3.1 and KCa2.3
Source: PLoS One. 2013 Mar 14;8(3):e58614. doi: 10.1371/journal.pone.0058614 (PMC3597730; doi:10.1371/journal.pone.0058614)
Supplement: Table S3 — 13b and SKA-31 did not modulate endothelium-independent relaxation to the NO donor, SNP, in porcine coronary artery pre-contracted by 60 mM K+. (PDF) [file pone.0058614.s007.pdf]

Table S3: 13b and SKA-31 did not modulate endothelium-independent relaxation to the NO donor, SNP, in porcine coronary artery pre-contracted by 60 mM K^+.

Compound(s)	n	% relaxation to 10 μM SNP	*P* vs. Ve
Vehicle (Ve)	7	85±6	
13b 0.5 μM	8	84±4	n.s.
SKA-31 1 μM	4	82±8	n.s.
SKA-31 10 μM	4	80±5	n.s.
13b 0.5 μM + SKA-31 1 μM	4	77±4	n.s.
13b 0.5 μM + SKA-31 10 μM	4	77±5	n.s.

Data are given as mean ± SEM; n.s. not significant.
